# Supervision of professionals: Interdependency between embodied experiences and professional knowledge

**DOI:** 10.3402/qhw.v10.28432

**Published:** 2015-11-17

**Authors:** Aud Marie Øien, Inger Johanne Solheim

**Affiliations:** Faculty of Social Sciences, Sogn and Fjordane University College, Sogndal, Norway

**Keywords:** Interdisciplinary supervision, professionals, action research, Norwegian psychomotor physiotherapy, phenomenology of perception, self-knowledge

## Abstract

Social work counsellors, exposed to hardships of clients’ lives, might, over time, experience strain as bodily reactions of muscle tension and pain. Within the framework of improving professional practice, the aim was to explore meanings attached to moving and breathing by studying the influence of supervision, encompassing experiences and reflections on bodily exercises, and reflection on challenging professional experiences. Action research of interdisciplinary supervision for seven counsellors, based on observations, field notes, reflection notes, and a focus group interview, was carried out. Data were analysed across participants within sessions and over time to compare meaning variations. The counsellors’ change of experiences were identified as phases: What is in it for me, not knowing what to perceive, attention as basis for knowing how to move, experiencing and creating connections, and knowing oneself better. Adjusted to change of experiences, supervisors encouraged counsellors to give attention to, become aware of, and relieve and explore muscle tension and breathing restrictions to contexts of meaning. Supervision based on movement opened access to personal learning. Supervision as approaches of movements and reflections contributed to increased self-knowledge in professional social work practices. Based on ability to perceive and relieve muscle tension and flow of breathing, the approach might be a potential for professionals to handle challenging situations. The findings, related to the lived body, encompass appearances of new meanings and new uses to experiences of muscular tension and flow of breathing.

Supervision in professional practice is part of developing professional knowledge (Davys & Beddoe, 2010). Social work counsellors point to supervision as crucial for professional endurance, particularly for the feeling of one's own well-being (Chiller & Crisp, 2012). Their exposure to clients’ hardship of life may over time be emotionally exhausting (Russ, Lonne, & Darlington, 2009). Health professionals increase their risks of strain and exhaustion on the basis of heavy work load, emotional reactions linked to patients’ problems, and conflicts among professionals (Hoskins, Coleman, & McNeely, 2005). Strain influences negatively the experiences of well-being and health, because of painful bodily reactions from muscular tension, restricted patterns of breathing, and the feelings of bodily detachment (Øien, 2010; Øien, Råheim, Iversen, & Steihaug, 2009). Supervision in social work mainly emphasizes reflection on professional management between less experienced supervisees and more experienced supervisors (Davys & Beddoe, 2010), but there is a growing interest in exploring bodily reactions based on reflection (Aubert, 2010; Tveiten, 2008). A report from vocational training of supervisors pointed to how the practice of yoga and meditation may improve skills to sustain attention on clients (Schure, Christopher, & Christopher, 2008). However, the understanding of how long-term group supervision utilizing bodily approaches might intervene in social work counsellors’ professional practice remains incomplete. In a previous article, we explored the influence of a bodily exercise approach on critical reflection of social counsellors’ professional practice (Solheim & Øien, 2014). The counsellors increased awareness and acceptance of their own bodily reactions, as well as critical reflection on demanding situations with clients, and to a minor degree with collaborators. In the present article, within the framework of improving professional practice for counsellors in social work, we aim to explore and describe meanings attached to movement and breathing by studying the influence of supervision, over time, encompassing experiences and reflections on bodily exercises and reflection on challenging professional experiences.

## The supervision program

The 13-session program, with each session lasting 2 h, consisted of two approaches. The first, reflection on experiences of practiced movements and breathing, intended to increase awareness of bodily reactions of strain and to vary movements, balance, and breathing. The second, reflection based on colleague supervision and roleplaying, aimed to enhance critical reflection on professional management of challenging situations. In this article, we mainly emphasise the first approach.

The supervisors, the authors, had extensive clinical experiences with clients suffering from psychological, physical, and social strain. The first author, a psychiatric physiotherapy specialist, was in charge of the exercise sequences, consisting of approximately 10 different movements. In addition to general verbal instruction, the physiotherapist adapted instructions to each person's pattern of moving and breathing, using specific guidance by touch that is hands-on. The second author, a specialist in clinical social work, was in charge of the reflection sequences on professional practice. Both participated in and observed all supervision sessions.

The selected movement approach, developed from the Norwegian PsychoMotor Physiotherapy perspective (NPMP), is based on the understanding of the body as a social phenomenon of lived experiences and a biological entity (Øien, 2010; Øien et al., 2009). Social, psychological, and physical strain influences the body by affecting the interdependent functions of moving, balancing, and breathing. Breathing interplays with both movements and emotions (Braatøy, 1952; Thornquist & Bunkan, 1991). The NPMP approach intends to readjust movements of the whole body, by use of relaxing and balancing exercises integrated to the rhythm of breathing (Øien, 2010; Øien et al., 2009; Thornquist & Bunkan, 1991). Standing in good balance includes giving weight to the middle of the feet, and straightening relaxed the body as a whole (Langaas, 2013; Thornquist & Bunkan, 1991).

## Theoretical perspective—the perceiving body

The study was mainly guided by the perspective of NPMP as described above, and phenomenology of perception, stating that our understanding is based on how our body, defined as subject, perceives the world (Merleau-Ponty, 2012). Phenomenology of perception is the study of the appearance of being in consciousness i.e., becoming aware how things are making sense for us (Merleau-Ponty, 2012). Merleau-Ponty describes meaning, i.e., significance or sense of experiences, as the intuitive connection things have for us when we find and handle them practically. Phenomenology describes basic structures of experience and understanding from the first-person perspective (Merleau-Ponty, 2012). Perceiving means having a body, which in turn means inhabiting a world. To perceive is to be familiar with, deal with, and find our way in environments based on sensory and motor dimensions (Merleau-Ponty, 2012). Intentionality, defined as skilful bodily responsiveness and spontaneity in direct engagement with the world, is a mode of existence, that is, a way of being in the world (Merleau-Ponty, 2012).

## Methodology, design, and methods

The phenomenological perspective of Merleau-Ponty (2012) points to knowledge built on the subject's direct experiences of the world. The perspective emphasizes the world as meaning, including the transformation of perception from something indeterminate to something more determinate. Being able to capture the counsellors’ process of making meaning, the design was longitudinal.

### Design

We chose action research design, aiming to increase knowledge and improve practice by following Malterud's (1995, 2011) steps of cycle of action: identifying problems; summarizing previous experiences; determining aims of intervention; planning, describing and implementing action; and finally, redefining problems based on experiences. In the introduction chapter, we identified problems, summarized previous experiences, and determined aims of intervention on a more general basis. The supervision program chapter included steps of planning and describing implemented actions.

The study, monitored from January to June 2011, consisted of 13 supervision sessions, and a subsequent focus group interview.

### Participants, context, and recruitment

The volunteering participants, six female social workers and one male administrator from the Norwegian Labour and Welfare Service, had between 2 and 12 years of clinical experience. All followed the whole program except one woman, who left after session nine due to change of work.

During the supervision and/or research process, the supervisors/researchers and the counsellors partly cooperated in making decisions. The supervisors decided the supervision program as a whole, and the counsellors influenced the content by, for instance, expressing need for guidance of movements by touch, as well as making less sound exercises.

### Data collection

To grasp and evaluate complex and subtle nuances of experiences, we built analysis on following empirical data: transcripts of audio-recorded sessions, reflection notes, field notes, and transcripts of the audio-recorded focus group interview. We tape-recorded 12 of the 13 sessions of supervision. Because of technical problems, session nine was not tape-recorded. However, the supervisors/researchers wrote thorough accounts from that session. Within each session, we wrote short field notes, except the extended one in session nine, of observed patterns of movements and breathing, and the counsellors’ expressed experiences and reflections of their bodily reactions. Immediately after each session, we wrote a second field note that included the supervisors’/researchers’ discussions of experiences and observations from the session, comparisons with the prior ones, as well as minor adjustments of planned implemented actions for the next session. Altogether, we made 26 written field notes. During the supervision process period, each counsellor was supposed to write three reflection notes, one in the first, the middle, and the last part, based on open-structured questions of experiences, understandings, and handlings of bodily reactions in professional situations. All seven counsellors delivered their first reflection note to the supervisors/researchers in the first session, the second in the fourth, and the third in the thirteenth session. Four counsellors wrote more notes than expected; i.e., one delivered five extra, another three, and two one each. Altogether, the counsellors wrote 31 reflection notes. Subsequent to the 13 sessions of supervision, six counsellors participated in one focus group interview of 2 h, reflecting retrospectively on their experiences of movements and breathing from the supervision process as a whole, and their professional practices. Prior to the focus group interview we informed the counsellors that the intention was to deepen reflections on their specific themes of interest from the supervision process. The interview guide included the main themes: experiences, understandings, and handlings of bodily reactions over time in supervision and professional practice, and experiences related to the supervision program.

The second author transcribed the verbatim tape-recordings from the sessions and the focus group interview. The first author wrote field notes during the interview.

### Data analysis

Data from tape-recorded sessions, field notes, and reflection notes were progressively analysed for use in ongoing supervision and research. In accordance with the first steps of Malterud's (2011, 2012) analytic approach, systematic text condensation, we, after each session, repeatedly read previous and new material to get an impression of the whole process. Based on data from all the data sources of each counsellor during the supervision process, we identified and labelled meaning units, as for instance, sensing breathing in upper part of the chest. We grouped similar meaning units within superior themes, as for instance, way of breathing. Kvale's contexts of interpretation, i.e., self-understanding, critical common sense, and theoretical understanding, guided our next steps (Kvale, 1996, 2007; Kvale & Brinkmann, 2012). Within the context of self-understanding, we rephrased meaning units into condensed form. For each counsellor, we built two summaries of condensed meaning units, one developed from audio-recorded transcripts and field notes, and one from their reflection notes. We compared themes from the summaries across counsellors, within sessions, and over time. At the critical common sense level, the interpretation of statements of the investigated phenomena, and of the persons who made the statements, was included in contexts of more general knowledge. The first two contexts of interpretation are presented in the findings. The third context of interpretation included application of theoretical perspectives presented in the discussion.

The transcribed focus group interview was read as a whole. Then, we selected and separated text of relevance in meaning units. Furthermore, we followed above described procedure. Summaries of themes, including extended understanding from a retrospective perspective, are presented in findings, for instance, “movements facilitate learning to know oneself.”

### Ethical considerations

The study was registered at Norwegian Social Science Data Services. We informed participants by letter about the aim and the supervision approaches of the study. We affirmed voluntary and anonymous participation to the counsellors. Discussing the supervisors’ different roles within and across the context of supervision, the counsellors expressed no hesitation of participation.

## Findings

The counsellors’ experiences of bodily reactions changed during the programme. The supervisors, but also the counsellors, contributed to evaluation and adaptation of the supervision approaches. In line with this, findings are presented as phases: (1) What is in it for me, (2) Not knowing what to perceive, (3) Attention as basis for knowing how to move, (4) Experiencing and creating connections, and (5) Knowing oneself better. The phases included the following steps of the cycle of action on a concrete level. The first phase comprised the counsellors’ identified and summarized previous experiences and present problems, implemented action, and a slight redefinition of problems based on emerging experiences.

The second, third, and forth phases pointed to the last steps, i.e., over time varying implemented action in accordance with redefined problems. The final phase encompassed the last step, redefinition of the problem anew.

### What is in it for me?

In the first session, the counsellors’ reflection notes about bodily reactions from prior meetings with clients functioned as a baseline for new experiences to come. Nearly all counsellors described experiences of headache, stiffness and pain in neck, shoulder, back or stomach, as well as tiredness, sleeplessness, loss of energy, and lack of concentration. One described experiences of breathing superficially. All expressed feelings of frustration, anger, unpleasantness, uneasiness, resignation, or sadness. In contrast, some emphasized feeling pleasure and empathy.

Based on data from field notes, the counsellors expressed expectations to discuss challenging cases, and explore how physical reactions, distress, and irritation might hamper the ability to give good help. One underscored the need to reduce pain.

#### Movement practice

Based on field notes and transcribed audio-recordings, the first sequence of movement practice functioned as basic assessment grounded on observation of movement performance and shared experiences. The physiotherapist instructed mainly verbally, considering use of touching to be intruding.

In general, the assessment pointed to reduced ability to relax, based on the following observations: The counsellors were standing out of balance with weight on their heels, in contrast to the middle of their feet, and increased extension and muscular tension of the back and the neck. Movement variations, shifts between contracting and relaxing muscles of specific body parts, were restricted. Breathing rhythm was mainly restricted to the upper chest, in contrast, to letting breathing flow spontaneously to the whole chest.

#### Counsellors’ reflections

The supervisors encouraged the counsellors to share and reflect on bodily experiences, but the exchange was scanty. However, many shared jokes and laughter. Some wondered about the reasons for the movement selection. In the subsequent approach, being expected to reflect on professional challenging experiences, they engaged in discussions about organization matters more than reflections.

#### Supervisors’ reflections after the session

The supervisors perceived the counsellors’ laughter, jokes, and non-sharing of experiences, as expressions of being unfamiliar with paying attention to their moving and breathing body. Therefore, we decided to enhance time of movement sequences in order to improve perception of bodily reactions and functions.

### Not knowing what to perceive

From the second to the fifth session the first uncertain attempts to pay attention to the body dominated. The physiotherapist instructed all in common in order to establish common ground with regard to balance, variation between contracting and releasing muscles, and holding or letting breath flow. Occasionally, she guided each by touching and posing questions about specific movement and space of breath, while waiting for change to come into being.

#### Movement practice

All counsellors found it unusual and challenging to attend to their body. They expressed uncertainty whether muscles, for instance the diaphragm, were tense or relaxed. They struggled to sit and stand balanced, and to move relaxed, as for instance, flexing back and neck without using much effort. The majority expressed increased breathing space by sometimes including movements of diaphragm in contrast to only breathing in upper chest part. A few struggled to change their forced breathing pattern.

#### Counsellors’ reflections

All counsellors perceived their body slightly more attentively. However, they described no transfer of experiences from the movement sequences to professional situations. Reflecting on professional experiences, they hardly made any reference to the body. They described experiences of anger, insecurity, not being in control of situations but feeling powerless, cornered, or offended.

#### Supervisors’ reflections after the sessions

Based on the counsellors’ emerging experiences of becoming more aware of their body in the movement sequences, the supervisors agreed upon two main areas of interest for the next sessions. The physiotherapist intensifies guidance by touch to improve attention to and perception of own body. Furthermore, to facilitate attention to bodily reactions in professional situations, the supervisors, in the second approach, explore more deeply the described experiences of emotional strain and pain.

### Attention as basis for knowing how to move

The sixth through eighth sessions included minor changes of improving attention to and varying movements and breath. Expressions of dissatisfaction appeared.

#### Movement practice

Most counsellors found it less challenging to attend to their body, perceiving more clearly variations of muscle tension and the distinction between holding and letting breathing flow. However, the few struggling also experienced some variation. The counsellors gradually experienced improved balancing, but episodes of not being in balance occurred.

#### Counsellors’ reflections

Many counsellors described being more conscious of their bodily reactions during movement practices and professional situations. One described how she contracted her chest, trying to breathe more relaxed. In professional situations, some also described acting differently, as for instance, breathing more calmly in dialogue with a client crying. Although improvements took place slowly, supervision of session eight emerged critical, as many described feeling tired of the repetitious exercises. One of them strongly disliked doing breathing exercises combined with sound expressions. Some found the physiotherapist’s use of touch challenging. Discussing the topic thoroughly, the counsellors emphasised that touch enhanced perception of ways of breathing and muscle tension. Counsellors also questioned the advantage of reflecting on professional situations as long as they had no influence on their working frame. In contrast, they shared common experiences of becoming aware of bodily reactions in threatening professional situations.

#### Supervisors’ reflections after sessions

Based on the complaints, the supervisors reflected on their own eagerness to promote change by pushing too hard. The supervisors decided to finish sound exercises, but keep up with repetitions. In line with the counsellors’ expressed need, we emphasised individual supervision by touching.

### Experiencing and creating connections

From the ninth through thirteenth sessions, based on individual points of departure, the counsellors deepened the quality of movement and breathing as well as attention directed to their own body.

#### Movement practice

The physiotherapist guided each individually by touch. The counsellors moved more attentively, and laughed less. The majority of them improved their balance, and reduced tension of specific muscles. One described change of balance as “hanging from the ceiling to stand heavily on the ground.” Some still struggled to coordinate balance of upper and lower body parts, and use less power. However, they described the increased awareness of their moving pattern, as useful to change these habits.

The counsellors were gradually breathing less restrictedly, by extending their breathing to diaphragm and abdominal regions. They connected change of breathing to a more relaxed mode of attention: “Previously, my breathing was restricted. I really, had to be attentive. Today, I lied down without thinking about it, a pleasant and queer experience.”

#### Counsellors’ reflections

Compared to prior periods, all counsellors expressed the importance of repeating exercises. Reflecting on the movement sequence, they underscored in details how guidance by touch helped, as for instance: “I very much like the small adjustments. If I move roughly on the right track you help me to move more precisely. If I'm not on track you help me to search it.”

Reflection on experiences of movement and breathing gradually increased. Improving ability to relax, they reflected on inability to adjust use of muscle power to deep-rooted embodied habits, described by one as “to use oneself as sledgehammer.” Some felt unfamiliar with these reflections.

More than previously, they deepened their reflections on transferring movement experiences to professional situations. One described maintaining the professional dialogue and creating space for action by handling one's own uncertainty and anger by instructing oneself to lean back breathing. However, in professional encounters, many forgot to attend to themselves.

#### Supervisors’ reflections after the sessions

The counsellors’ had varied expression of meanings connected to their bodily experiences. Some counsellors experienced only minor changes of reflection on their own pattern of moving and acting. However, varying patterns of acting in professional practices was challenging. The supervisors reminded themselves of the counsellors’ need for time to acquire and express change in professional situations.

### Knowing oneself better

In reflection notes and the focus group interview, the counsellors shared how supervision based on movements, to a considerable extent, opened access to personal learning and development, that is, learning to know oneself in new and varied ways. One expressed: “This approach represents a new world, a new way to develop knowledge about oneself.” Another specifically stated that to move and give attention to the body is to learn who you are. In data from the focus group interview and field notes they stated that guidance by touch was indispensable to improve awareness and knowledge of the body. “You laid your hand on the diaphragm, perceived muscle tension and said: Let the breath come. There you relaxed. Did you feel that?” Some counsellors preferred the physiotherapist to stay by their side until they experienced a change. Others wanted more time on their own to explore emerging changes.

Data from field notes, reflection notes, and the focus group interview pointed to how exploring new ways of breathing, moving, and balancing facilitated knowledge of getting more hold of themselves. They described how the experience of breathing more easily created an inner experience of having breathing space. This experience, further described more specifically by one, as holding one's own space, opened possibilities to adjust one's own distance to clients, “to act as a crutch in contrast to carrying the client.” Recognizing the experiences, they all contrasted this professional attitude to being overinvolved and overwhelmed by feelings. Some clearly underscored how bodily-based knowledge helped them to distinguish between their own needs and feelings and the clients’. In line with this, one counsellor emphasised how becoming more aware of one's own bodily reactions, made it possible to tune in to challenging situations ahead of meeting clients or collaborators, and thus avoid feeling distressed afterwards. Hence, they described the approach as a tool to take care of oneself in busy daily work. They underscored the need for time to turn use of the tool into ongoing and embodied habits.

## Discussion

### Paying attention to the body

In the initial sessions a gap appeared between retrospectively describing bodily pain and relating to the body in the present moment of moving. The counsellors seemed to relate to themselves in inattentive ways. Only few expressed perceptions of their way of moving and breathing, or shared challenging professional situations. Hence, understanding oneself on the basis of being an embodied perceiver seemed to be beyond reach (Merleau-Ponty, 2012). Perception is the basis of experiences of subjectivity and objectivity, that is, inner feelings and outward grasp on the world (Merleau-Ponty, 2012). Committing themselves to acquire a cheerful atmosphere, the counsellors seemed to keep distance from painful bodily reactions and challenging professional situations. Grasping the outward world seemed easier than grasping one's own feelings. This way of acting and moving could be seen as acquired embodied habits developed while relating to colleagues and clients. In line with this, Knight (2012) found social workers’ attitudes towards and engagement in self-disclosure limited both in settings of supervision and colleague interaction. Ingram (2015) reported that informal contact with colleagues was a preferred forum for articulating emotional content of practice compared to supervision forum. In our study, the counsellors did select the supervision programme that emphasised exploring interdependent reciprocity between movements, breathing, and emotional expressions in contexts of supervision and profession. In NPMP treatment of patients with chronic muscle pain, time was required to become in touch with movements of varied muscle tension, and even more to embodied emotional experiences and expressions (Øien et al., 2009).

To go looking for something, we need to know what we are looking for (Merleau-Ponty, 2012). The essence of perception is the contrast between figure and background (Merleau-Ponty, 2012). The counsellors did not know how to explore local muscle tension, the figure, on the background of a general feeling of tension. Attention, the active constitution of a new object, develops and thematises what was until then experienced as an indeterminate entity (Merleau-Ponty, 2012). The supervisors contributed to learning, by emphasising the act of attention to grasp concrete local muscle tension and restricted breathing.

### Slowly becoming in touch with the body

In each session, supervisors facilitated self-attention by instructing verbally and non-verbally specific ways of moving and letting breathing flow, and by assisting reflection on bodily experiences. Being attentive is to create a perceptual field by determining the location of a point of the body through, for instance, touch (Merleau-Ponty, 2012). Moving more attentively, the counsellors contributed to experiences of tensed muscles and restrained breathing, figures contrasted to indeterminate background of vague general tension. Movements and perceptions of movements influence each other reciprocally (Merleau-Ponty, 2012). Like perceiving, moving is a manner of relating to an object (Merleau-Ponty, 2012). Also, perception and attention are reciprocally interdependent. In initial sessions, the counsellors gradually became conscious of their restrained patterns of moving and breathing. Thus, possibilities opened to explore how to vary these patterns within settings of supervision and challenging professional situations. Consciousness, defined as self-knowledge, is grasped in the act of learning, in mutual dependence on perception and attention (Merleau-Ponty, 2012). The counsellors’ nascent self-knowledge seemed to be in line with an immediate self-consciousness that involves senses of ownership and agency (Gallagher, 2000). The counsellors, sensing their restricted space of breathing, implicitly experienced their breathing body, that is, self-ownership. Their sense of agency appeared the moment they experienced the action of, for instance, varying breathing by breathing more relaxed.

### Acquiring new uses of the body

In the last part of the supervision program, new ways of using the body emerged. The counsellors paid attention to their body with less effort. Perceiving imbalance, restrictions of breath, and muscle tension, they gradually acquired more varied and relaxed ways of moving, balancing, and breathing within and partly beyond the moving sequences. These findings correspond to clinical studies of change during physiotherapy for patients with chronic muscle pain (Øien, Iversen, & Stensland, 2007; Øien et al., 2009). The counsellors described change of moving from using too much muscle power of upper body parts, to giving more weight to the feet, perceived by one as “hanging from the ceiling light to stand heavily on the ground.” Establishing new habits of moving alongside the prior ones seemed to take place. These new-appearing motor and perceptual habits could be understood as acquisition of a new world or mode of existence, that is, new ways of being or acting in situations (Merleau-Ponty, 2012). Describing change of moving, the counsellors gave meaning to their experiences. In accordance with Merleau-Ponty (2012) new meaning is created when previous movements are integrated into new motor entity. When the counsellors sensed and partly used varied muscle power without losing their foothold, the first sign of understanding themselves as slightly changed appeared. Developed over time, these emerging experiences actualized the concept of the narrative self, including memories of the past and intentions toward the future (Gallagher, 2000). Self-knowledge appeared not only as consciousness of moments of present experiences, but also as emergent experiences developed over time.

The participants reflected on their way of moving and breathing. Reflection as creativity participates in materialising what is until now not-reflected (Merleau-Ponty, 2012). In the concrete act of reflection, the counsellors learned to know how indeterminate experiences of breathing gradually became determinate perceptions of holding breath. Knowing oneself, an individual begins to reflect upon oneself in contrast to objects outside (Merleau-Ponty, 2012). Reflecting, the counsellors’ nascent exploration of making different use of the body emerged. Some emphasised how they in professional situations of anger and uncertainty, facilitated self-relaxation by leaning back and breathing more deeply. Others experienced how their body came into use as emotional expressions in threatening professional situations. Expressions and meanings are connected to language (Merleau-Ponty, 2012). Implicitly, the counsellors extended their knowledge to include embodied emotional expressions in professional relationships. However, few reflected on the reciprocal exchange of bodily expressions as communication, and thereby, as constituents of relationships.

### The body as means for developing self-knowledge

Reflecting on the supervision as a whole, the counsellors confirmed knowledge based on perceptual appearances of bodily experiences. Perception is the source of knowledge (Merleau-Ponty, 2012). All counsellors emphasised how moving and paying attention to the body opened possibilities to develop knowledge of and about themselves. Among all, the experience of being more in control of oneself appeared as acquisition of knowledge in contrast to experiences from initial sessions of feeling powerless and uncertain. The findings indicated that the opportunity for choice may be a vehicle to enhance an individual's perception of control and self-efficacy (Leotti, Iyengar, & Ochsner, 2010). Furthermore, perception of control seems to be important for regulating emotional reactions in stressful situations (Leotti, Iyengar, & Ochsner, 2010). The NPMP perspective emphasizes the dependent reciprocity between ways of breathing and emotional experiences (Øien et al., 2009). The counsellors acquired possibilities to choose more relaxed ways of moving and breathing. One counsellor signified the experience of being more in control of herself as having her own breathing space, pointing to a relaxed way of breathing, in contrast to prior experiences of holding her breathing. The expression, having one's own breathing space, encompasses the biological movement of breathing and the movement of expression from the language world. The bodily event has a psychological signification (Merleau-Ponty, 2012). Here, the sign is inhabited by its meaning (Merleau-Ponty, 2012). The act of extending one's breathing space is accomplished in the professional relationship, signified as holding and taking care of one's own space towards the client. To hold one's space in professional relationships appeared as contrasts to feeling boundless, overinvolved, or overwhelmed by others’ feelings. The experience opened the potential to adapt to the client in more varied ways, for instance, by acting as crutch in contrast to carrying the other. The means of knowing the body, i.e., oneself, is by living it, that is, to be committed to specific situations (Merleau-Ponty, 2012). The counsellors gradually learned to be more present in specific situations of supervision and professional practice. Practising new acquisitions in professional situations with clients appeared as challenging but possible. Experiences of learning to know oneself in new ways indicated a potential to choose to take better care of oneself and to give clients possibilities to develop their own footholds.

### Trustworthiness and transferability

Reflexivity, validity, and relevance are essential standards for qualitative research (Malterud, 2001, 2011). Reflexivity is defined as thoughtful, self-aware analysis of intersubjective dynamics between researcher and the researched (Finlay & Gough, 2003). Reflexion on our double roles as researchers and supervisors took place at all stages. Meta-positions are strategies for creating adequate distance from a study setting that you are personally involved in (2001, 2011). Prior and subsequent to each session, we respectively, planned focus of attention, and posed critical questions to our interpretation of observations. Within sessions, we alternated between supervising actively, and writing field notes. The validity of clinical evidence can be strengthened when different perspectives complement each other (Malterud, 2001, 2011). The first author's knowledge of perspectives of movements and breath was decisive to assess the counsellors’ acquired bodily knowledge, whereas the second author's knowledge of the professional field of the research context contributed to understand the complexity of the counsellors’ tasks. The researchers’ different professions involved nearness and distance to the research context, and opened for critical reflexion on emerging construction of knowledge. The aim of triangulation is to increase the understanding of complex phenomena (Malterud, 2001, 2011). We applied triangulation of researchers and methods. The value of the produced knowledge should be evaluated with regard to relevance and practical use (Kvale, 2007; Malterud, 2001; Lyons & Coyle, 2007). Communicating findings to counsellors in professional and research contexts, beyond the study context, gave rise to recognition. The findings might be transferred to supervision contexts of other clinical professions if our path to knowledge is judged trustworthy. The findings are in line with studies of long-term NPMP treatment of patients with chronic muscle pain (Øien, 2010; Øien et al., 2009).

## Conclusion and implications for practice

This study describes how supervision of social work counsellors, in a broader framework than normally practised, contributed to increased self-knowledge in professional practices. The major implication of this study is to include a movement approach to explore and facilitate change of restrictions of muscle tension, imbalance, and breathing. Based on Merleau-Ponty's perspective of the body as embodied subject, attention, guidance by touch, and reflection on functions of moving and breathing gradually improved self-knowledge in contexts of supervision and professional situations. This enhanced a change of restricted muscle tension, imbalance, and breathing, as well as the feeling of not being in control. In challenging professional situations bodily-based self-knowledge might be a potential to vary professional-client relationships by turning bodily restrictions to bodily resources.

## Ethical approval

Norwegian Social Science Data Services was informed about and approved the project, reference 24504. The Regional Committee for Medical Research Ethics, Health Region West, Norway, reference 2252, date 23.09.2010, assessed the project to be carried out and the results to be published without acceptance from REK, because collected data was not to be considered as health information. The research study includes professionals’ experiences of their own reactions from contexts of supervision and professional situations with clients.
